# Combining multiplex gene editing and doubled haploid technology in maize

**DOI:** 10.1111/nph.19021

**Published:** 2023-06-12

**Authors:** Lennert Impens, Christian D. Lorenzo, Wout Vandeputte, Pieter Wytynck, Kevin Debray, Jari Haeghebaert, Denia Herwegh, Thomas B. Jacobs, Tom Ruttink, Hilde Nelissen, Dirk Inzé, Laurens Pauwels

**Affiliations:** 1 department of Plant Biotechnology and Bioinformatics, Ghent University, B-9052 Ghent, Belgium; 2 Center for Plant Systems Biology, VIB, B-9052 Ghent, Belgium; 3 Flanders Research Institute for Agriculture, Fisheries and Food (ILVO), B-9820 Merelbeke, Belgium

**Keywords:** CRISPR/Cas9, doubled haploids, gene editing, gene family, haploid induction, maize, multiplex gene editing, mutation stacking

## Abstract

- A major advantage of using CRISPR/Cas9 for gene editing is multiplexing, i.e. the simultaneous targeting of many genes. However, primary transformants typically contain hetero-allelic mutations or are genetic mosaic, while genetically stable lines that are homozygous are desired for functional analysis. Currently, a dedicated and labor-intensive effort is required to obtain such higher-order mutants through several generations of genetic crosses and genotyping.
- We describe the design and validation of a rapid and efficient strategy to produce lines of genetically identical plants carrying various combinations of homozygous edits, suitable for replicated analysis of phenotypical differences. This approach was achieved by combining highly multiplex gene editing in *Zea mays* (maize) with *in vivo* haploid induction, and efficient *in vitro* generation of doubled haploid plants using embryo rescue doubling.
- By combining three CRISPR/Cas9 constructs that target in total 36 genes potentially involved in leaf growth, we generated an array of homozygous lines with various combinations of edits within three generations. Several genotypes show a reproducible 10% increase in leaf size, including a septuple mutant combination.
- We anticipate that our strategy will facilitate the study of gene families via multiplex CRISPR mutagenesis and the identification of allele combinations to improve quantitative crop traits.

## Introduction

Gene editing is the preferred tool for the creation of novel alleles for plant genetic research and breeding (Zhu *et al.,* 2020). Subsequent phenotypic analysis is used to associate genes to (molecular) functions and traits such as nutritional quality, yield, and resistance to (a)biotic stress (H.-J. Liu *et al.,* 2020; Lorenzo *et al.,* 2023). Because drought and heat or salt stress are likely to become more important due to climate change, plant responses to these abiotic stresses are intensely studied to improve agricultural production and contribute to the sustainability of the global food system (Verslues *et al.,* 2023). Many of these plant traits are controlled by a complex interaction between many different genes and require the identification of the right combinations of alleles to have pronounced and desired effects (Vanhaeren *et al.,* 2014; Vanhaeren *et al.,* 2016; Sun *et al.,* 2017). Having the right combinations of alleles is also valuable in overcoming genetic redundancy, which is common in plants (Corcoran *et al.*, 2022). Hence, the ability to stack multiple gene edits in the same plant is crucial for the engineering of complex traits. Multiplexing, i.e. the simultaneous targeting of many genes, is one of the major advantages of the Clustered Regularly Interspaced Short Palindromic Repeats/CRISPR associated protein 9 (CRISPR/Cas9) gene editing system. Expression of the Cas9 protein can be combined with multiple guide RNAs (gRNAs), only differing in the spacer sequence and targeting a variety of genes, including multiple members of gene families (Lorenzo *et al.*, 2023). However, one limitation of this approach is that primary transformants typically contain hetero-allelic mutations and/or show genetic mosaicism, i.e. a tissue will show more alleles than its ploidy level. Because each genetically unlinked new allele segregates independently, subsequent generations show a large mixture of edited allele combinations in homozygous and/or heterozygous states. Moreover, new alleles may occur in these subsequent generations when the CRISPR/Cas9 system remains present, termed transgenerational gene editing (Wang *et al.*, 2018). This abundance of segregating alleles and novel editing can be used as a rich source for breeding but also complicates associating genotypes to phenotypes (Lorenzo *et al.*, 2023). To obtain genetically stable mutant lines for replicated phenotyping in multiple environments, homozygosity is desired at all edited loci (Ingvarsson & Street, 2011). Obtaining such lines by Mendelian segregation requires a labor-intensive effort of iterative rounds of self-pollination and selection over many generations, which becomes increasingly more difficult with increasing numbers of target genes. For example, with six different independently segregating loci containing heterozygous mutations, only ${\left(\frac{1}{4}\right)}^{6}$ of the progeny after a self-cross are expected to be homozygous for all targeted loci. If we use a probability of 90% to obtain at least one such plant, 9430 plants would need to be screened (Fig. S1, (Lübberstedt & Frei, 2012)). This is an unattainable strategy to generate highly edited homozygous plants, especially when greenhouse space is limited. With maize, having a generation time of 3 to 4 months, recurrent self-crossing and genotyping is a tedious, resource-intensive, and time-consuming strategy.

*In vivo* haploid induction has been an indispensable tool for hybrid breeding strategies to create uniform homozygous inbred lines. *In vivo* haploid induction is the process of uniparental chromosome elimination during early embryogenesis after inter- or intraspecific hybridization. For example, wheat haploids can be produced by pollination of emasculated wheat spikes with maize pollen in an interspecific cross (Devaux, 2021). Alternatively, *in vivo* haploid inducer (HI) lines produce haploid progeny after an intraspecific cross. In maize, HI lines produce a certain percentage of haploid progeny containing only the chromosomes of the mother (maternal HI lines) or the father (paternal HI lines). Since the discovery of the stock 6 inbred line with a haploid induction rate (HIR) of 1-3% (Coe Jr, 1959), several other maternal HI lines such as RWS have been derived from stock 6 with an even higher HIR (Röber *et al.,* 2005; Chang & Coe, 2009; Xu *et al.,* 2013). More recently, the underlying mechanism of maize maternal haploid induction was discovered by the mapping of a quantitative trait locus that pinpointed a 4-bp insertion in *MATRILINEAL/NOT LIKE DAD/ZmPHOSPHOLIPASE-A1 (MTL/NLD/ZmPLA1),* a pollen-specific gene encoding a patatin- like phospholipase A (Gilles *et al.,* 2017; Kelliher *et al.,* 2017; Liu *et al.,* 2017). Later, a knockout mutation in *ZmDMP* (encoding a DUF679 membrane protein) was found to have a synergistic effect on HIR when combined with *mtl* (Zhong *et al.,* 2019). In combination with an effective method to discriminate haploids from diploids (haploid identification) and a subsequent method for genome doubling, modern HI lines with a high HIR are a great resource for the fast production of homozygous lines.

Here, we report Gene Editing followed by Doubled Haploid production (GEDH), a strategy to combine multiplex gene-edited (GE) maize plants with *in vivo* haploid induction, and subsequent *in vitro* generation of doubled haploid (DH) plants using embryo rescue doubling. This combined approach allows the efficient production of fixed GE lines in only three generations, with each line having a combination of homozygous edits suitable for phenotyping experiments.

## Materials and Methods

### Plant material, handling, and growth conditions

Seeds of the *Zea mays* (maize) inbred line B104 were originally obtained from the USDA National Plant Germplasm System (Accession no. PI 594047). Single maize B104 seeds were placed in a pre-wetted Jiffy-7^®^ pellet and kept in controlled growth room conditions (300 μE.m^-2^.s^-1^, 16 h light (23°C), and 8 h dark (22°C)). The seedlings were transferred to medium-sized pots with professional potting mixture (Van Israel nv). After three weeks, plants were transferred to 10-L pots with professional potting mixture (Van Israel nv) containing controlled release fertilizer (2.0 kg.m^-3^, Osmocote^^®^^, NPK 12/14/24) and moved to the greenhouse (16 h light (25°C), and 8 h dark (22°C)) until grown to maturity. Seeds of the RWS-GFP line, expressing Green Fluorescent Protein (GFP), were originally obtained from the lab of Dr. James Birchler (Yu & Birchler, 2016). RWS is a descendant of a cross between the Russian line KEMS and WS14 (Röber *et al.,* 2005). Because the time from sowing until flowering is 70 days for B104 and 60 days for RWS-GFP, the time of seed sowing was adjusted accordingly to ensure that parent (edited) B104 ears could be pollinated by RWS-GFP pollen, in our greenhouse. Emerging ears of maize plants were covered with a paper shoot bag to avoid unwanted cross-pollination. Silks of B104 female flowers were cut back (3-5 cm from the top, without nicking the ear itself) one day before pollination. On the day of pollination, a paper tassel bag was used to collect RWS-GFP pollen. Anthers and pollen were separated by shaking the bag gently and pollen was sprinkled onto the re-emerged silks of the parent B104 plant.

### Embryo isolation and colchicine treatment

Fourteen days after pollination, cobs were harvested from the plants, husk leaves were removed and the cobs were surface-sterilized in 5% NaOCl and 0.01% Tween^®^ 20 (Sigma- Aldrich) solution for 2 min. Sterilized cobs were washed three times with sterile purified water. A sterile scalpel was used to cut off the top of the kernels and a small sterile spatula was used to isolate all embryos from the cob. Embryos were all collected on a square Petri dish with basic plant medium (Regeneration II medium (Aesaert *et al.*, 2022)). This medium was also used for colchicine treatment and subsequent germination of the embryos. 1 L medium is composed of 4.3 g Murashige and Skoog (MS) salts, 30 g sucrose, 100 mg myoinositol, pH 5.8, then 3 g gelrite was added and the medium was autoclaved. After autoclaving, 1 mL of a 1000x stock of MS vitamins was added. Colchicine treatment of embryos was based on the method described by Barton *et al.* (2014) with modifications. Fluorescent microscopy was used to separate GFP-expressing diploid embryos from non-GFP-expressing haploid embryos. Diploid embryos were discarded and haploid embryos were moved onto plates with Regeneration II plant medium, supplemented with 0.05% colchicine and 0.5% dimethylsulfoxide (colchicine was dissolved in dimethylsulfoxide and the mixture was added to the medium after autoclaving). Haploid embryos were put with the scutellum facing upwards onto the colchicine-containing medium spaced at least a few mm apart and incubated for 24 h in the dark at 25°C. Treated embryos were moved to Sterivent high containers (107x94x96 mm, Duchefa, Haarlem, The Netherlands) containing Regeneration II medium without colchicine and placed with the scutellum facing down. Embryos were incubated for approximately six days in the dark at 25°C; etiolated roots and shoots emerge from the embryos at this stage. After six days, the Sterivent containers were moved to light conditions (80-100 μE.m^-2^.s^-1^, 16 h light (24°C), and 8 h dark (22°C)) where they grew into green plantlets. The plantlets were then transferred to a pre-wetted Jiffy-7^®^ pellet and covered with a plastic box to maintain high humidity; facilitating the transition from tissue culture to the growth chamber. Plantlets were kept in controlled growth room conditions (300 μE.m^-2^.s^-1^, 16 h light (25°C), and 8 h dark (22°C)). The humidifying cover was removed after three days. After 2-3 weeks, plantlets were tested by flow cytometry. Selected plants were transferred to larger pots and moved to the greenhouse until maturity.

### Flow cytometry analysis

Flow cytometry analysis was used to assess the ploidy levels of the colchicine-treated plants. Approximately 1 cm^2^ of leaf tip material (leaf 3-4) was cut from the plants and chopped into fine pieces using a razor blade in 200 μL chilled CyStain UV Precise P Nuclei Extraction Buffer (Partec) and supplemented with 800 μL chilled CyStain UV Precise P Nuclei Staining Buffer (Partec). The mixture was filtered through a 50-μm filter and analyzed with a CyFlow^®^ML cytometer (Partec). An untreated, diploid plant was used as a control. The DNA content distribution of the nuclei was analyzed using FloMax (Windows™) and/or the Floreada web tool (https://floreada.io/analysis).

### Genomic DNA isolation and multiplex amplicon sequencing

A piece of 1-2 cm of leaf material was placed in 8-strip, 2-mL capacity tubes (National Scientific Supply Co) together with two 3-mm stainless steel ball bearings, snap frozen in liquid nitrogen, and ground using a Mixer Mill MM400 (Retsch^®^). 0.5 mL DNA extraction buffer (2.5 mL 1 M Tris-HCl pH 8, 3 mL 5 M NaCl, 5 g saccharose, to 50 mL with Milli-Q water) was added, and samples were shaken and incubated at 65°C for 20 min. Tubes were centrifuged (2 min at 1800 x g) and 50 μL of supernatant was mixed with 70 μL magnetic beads (HighPrep™ PCR Clean-up System, Magbio) and put on a magnet. The supernatant was taken off, beads were washed twice with 80% ethanol and dried for further processing. Highly multiplex amplicon sequencing (HiPlex, Floodlight Genomics LLC, Knoxville, TN, USA) was performed as described in Lorenzo *et al.* (2023).

### Numerical calculations

A specific homozygous edit combination has an expected frequency $p=(\frac{1}{4}{)}^{g}$ after a self-cross and $p=(\frac{1}{2}{)}^{g}$ after haploid doubling of a heterozygous parent, with *g* the number of unlinked edits considered (Fig. S1a, Lübberstedt and Frei (2012)). The minimal population is $n_{\min}\geq \frac{\ln(1-Q)}{\ln(1-p)}$ with *Q* the probability of finding at least one such plant (Fig. S1b). For example, finding a specific combination of six edits with 90% probability requires genotyping 146 vs 9430 plants using haploid doubling and self-crossing, respectively. To create Fig. S1b new equations were derived maintaining the naming convention. With *n* the (minimal) number of plants: $n=\frac{\ln(1-Q)}{\ln(1-p)}$

$$\begin{array}{c} \Leftrightarrow \ln(1-Q)=n\times \ln(1-p)\\ \Leftrightarrow (1-Q)=e^{n\times \ln(1-p)}\\ \Leftrightarrow Q=1-e^{n\times \ln(1-p)} \end{array}$$

### Phenotyping

Phenotyping analysis was performed as described in Lorenzo *et al.* (2023). For the phenotypic experiments, only well-watered conditions were used (2.4 g of water per gram of dry potting mix, replenished three times a week). Plants were grown in the growth chamber (23°C) under a long-day photoperiod (16 h : 8 h, light : dark). Phenotypic traits were measured for all plants at V3 (when the collar of leaf 3 was fully developed). Final leaf 3 length (FLL3) was measured from the crown of the plant to the leaf tip and final leaf 3 width (FLW3) was measured at the widest point of the leaf blade. Pseudo leaf area (PLA3) was calculated based on other measurements: PLA3 = FLL3xFLW3. All statistical tests were performed at a significance level of 5%. Normality was assessed using the Shapiro-Wilk Normality test and Q-Q plots. The equality of variances was studied with Levene’s test. The Kruskal-Wallis rank sum test was used to find if any of the groups was different from another. A significant result of the Kruskal-Wallis rank sum test was followed by multiple pairwise Wilcoxon rank sum tests to detect which groups were different from each other (multiple testing correction was performed using the Holm correction (Holm, 1979)). Data analysis was performed using R (version 4.0.3) with the packages; *stats* and *car* (figures were made with ggplot2).

## Results

### Efficient maize haploid induction and embryo rescue doubling using the B104 inbred line

**Fig. 1:**
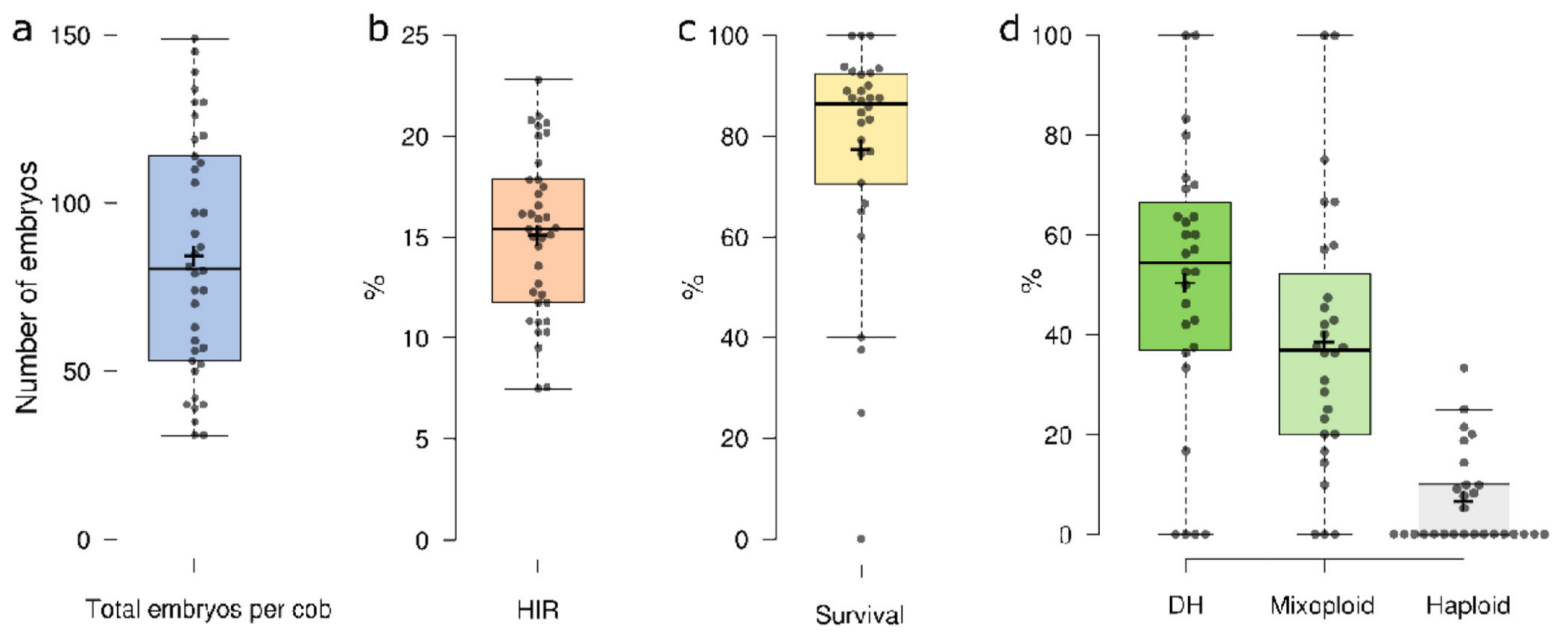
Efficient haploid induction and haploid doubling in Zea mays (maize) B104. (**a**) Number of embryos obtained per cross between B104 background (female) and RWS-GFP (male) (n = 38 independent pollinations). (**b**) Haploid induction rate (HIR). Percentage of embryos scored as haploid based on the absence of GFP from a cross between B104 background and RWS-GFP (n = 38). (**c**) Percentage of haploid plants in each independent experiment surviving the embryo rescue doubling process (n = 30). (**d**) Haploid doubling rate. Percentage of plants in each experiment scored either as doubled haploid (DH), mixoploid, or haploid based on flow cytometry analysis of leaf 3 or 4, calculated per treated haploid embryo (n = 28). Boxplots with jittered data points; center lines show the medians; box limits indicate the 25^th^ and 75^th^ percentiles; whiskers extend 1.5 times the interquartile range from the 25^th^ and 75^th^ percentiles, and the mean is indicated as a cross.

We used the HI line RWS-GFP, which contains a GFP fluorescent marker under the control of the CaMV 35S promoter (Yu & Birchler, 2016) as a pollen donor. As *in vivo* haploid induction by RWS functions by paternal genome elimination, haploid embryos can be identified by scoring the absence of GFP fluorescence. We first examined haploid induction using the public maize inbred line B104, which is often used for transformation and gene editing by the research community (Frame *et al.,* 2006; Aesaert *et al.,* 2022; Kang *et al.,* 2022), as the maternal donor. In addition to the HI genotype, the maternal donor genotype together with environmental conditions also influences the HIR (Kalinowska *et al.,* 2019). In 38 independent crosses between B104 (female) and RWS-GFP (male), we obtained on average 84 embryos per cross (Figs 1a, S2a), of which 15.1% were haploid based on the absence of GFP fluorescence (Figs 1b, S2b). Because maize haploid plants are sterile, their genomic content needs to be doubled to generate fertile diploids, i.e. doubled haploids (DH; DH0 is the first DH generation, DH1 its progeny after self-pollination). Colchicine-mediated doubling of maize haploid seedlings is routinely used in large-scale DH breeding programs, but because only 10-30% of seedlings yield DH1 seed, it is not very efficient (Chaikam *et al.,* 2019; Molenaar *et al.,* 2019). These routine strategies require whole or partial submersion of seedlings in a colchicine solution, which complicates the containment and safe handling of this toxic chemical. To simplify and quicken the doubling procedure, we used embryo rescue doubling (ERD), a method that combines embryo rescue with colchicine treatment (Barton *et al.*, 2014; McCaw *et al.,* 2021). Haploid immature maize embryos were placed *in vitro* on basic plant medium containing colchicine followed by germination. On average, 78.4% of isolated B104 embryos survived ERD and germinated *in vitro* (Fig. 1c). After transferring germinated plantlets to the growth chamber, flow cytometry analysis using material of the third or fourth leaf showed that, on average, 55.4% of plants were diploid and an additional 36.8% were mosaic for doubling (mixoploid) (Figs 1d, S3). Haploid reproductive cells cannot undergo meiosis, therefore, gametes will not be derived from haploid tissue (Chalyk, 1994). Mixoploid plants resulting from incomplete haploid doubling have a mixture of haploid and diploid tissue, but any gametes will be derived from diploid tissue (Chaikam *et al.*, 2019). We retained all plants scored as non-haploid (DH0) for future experiments. In conclusion, we were able to set up an efficient pipeline for haploid induction in B104 by crossing with the RWS-GFP HI line, followed by ERD, yielding on average 9.6 DH0 plants per cross.

### GEDH, a strategy to combine multiplex gene editing and doubled haploid breeding

**Fig. 2:**
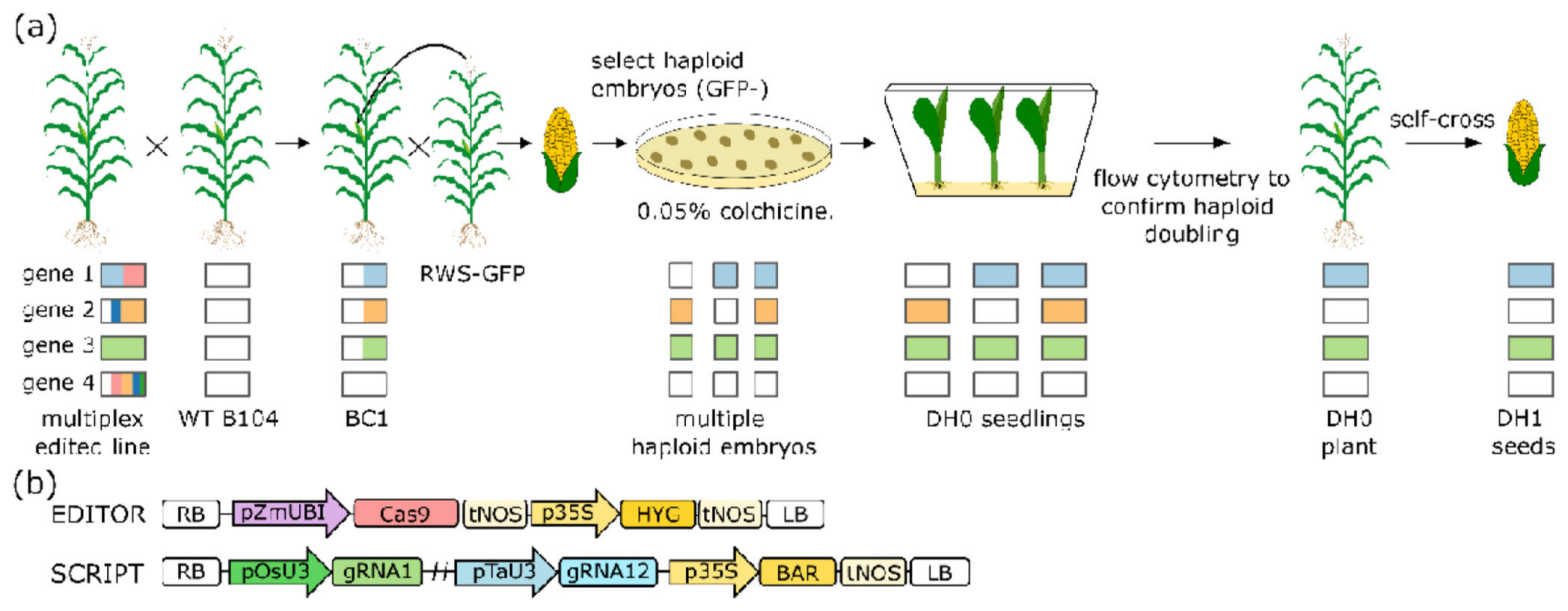
Combining multiplex gene editing and doubled haploid breeding in Zea mays (maize). (**a**) Overview of the GEDH strategy. For simplicity, four GE target loci are shown to represent a genotype, stacked colored bars represent different edited alleles, and white bars indicate reference alleles, bar lengths are proportional to the allele frequencies. More than two alleles can be present in a plant due to genetic mosaicism from ongoing Cas9 activity. Multiplex edited T0 plants are crossed with wild-type (WT) B104 plants resulting in BC1 plants that are either heterozygous or wild-type at each locus and can be selected for the absence of the EDITOR T-DNA to avoid further editing. BC1 plants are pollinated using a haploid inducer line carrying a GFP transgene (RWS-GFP). Two weeks after pollination, embryos are isolated, haploid embryos are selected for the absence of GFP fluorescence (GFP-), and incubated on a medium containing colchicine. Colchicine-treated embryos are germinated *in vitro* and chromosome doubling is confirmed by flow cytometry. Every haploid embryo and doubled haploid plant will show a random combination of edited loci. Doubled haploids (DH0) are self-pollinated to obtain genetically identical homozygous DH1 seeds. (**b**) Diagrams of the EDITOR and SCRIPT T-DNAs. RB, right border; pZmUBI, maize *UBIQUITIN-1* promoter; tNOS, Agrobacterium *NOPALINE SYNTHASE* terminator; p35S, double Cauliflower mosaic virus 35S promoter; HYG,*HYGROMYCINE RESISTANCE;* BAR, *BIALAPHOS RESISTANCE,* LB, left border. In the SCRIPT T-DNA, 12 gRNAs are alternately expressed by either an *OsU3* or *TaU3* promoter.

**Fig. 3:**
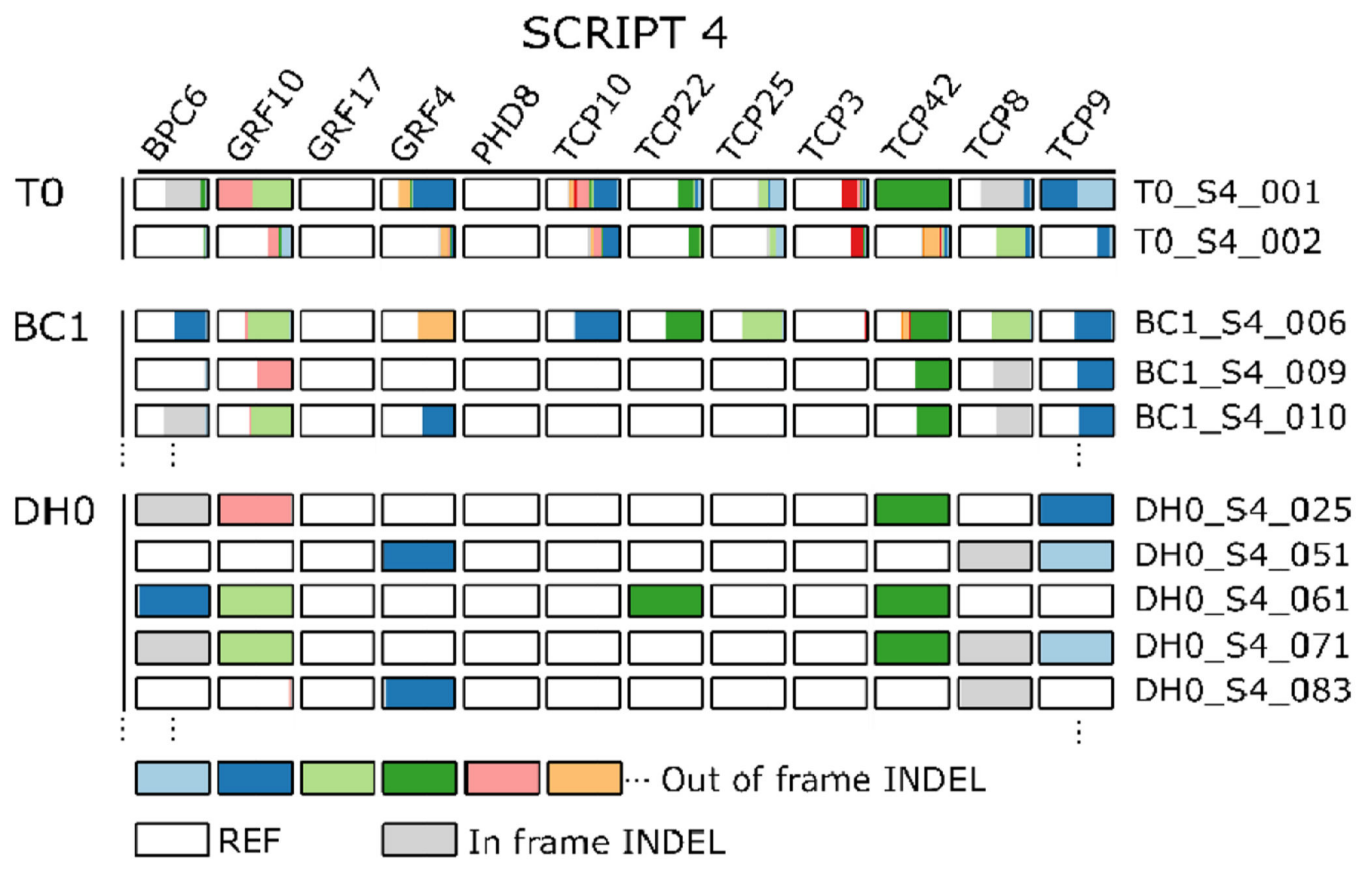
Combining haploid induction and multiplex gene editing in *Zea mays* (maize) with SCRIPT 4. Maize B104 immature embryos heterozygous for the EDITOR T-DNA were supertransformed with the SCRIPT 4 gRNA construct, targeting the 12 genes listed on top. Experimentally obtained genotypes for a representative subset of plants are shown for each generation. Colored, horizontally stacked bars each indicate different mutant out-of-frame alleles per target locus, white bars indicate wild-type (reference) alleles, light gray bars indicate in-frame mutant alleles and colored bar lengths are proportional to the fraction of sequence reads per locus containing the allele. Bar lengths <50% are indicative of mosaicism.

To evaluate the concept of combining DH technology and multiplex gene editing (Fig. 2a), we used multiplex CRISPR/Cas9 genome-edited B104 maize plants obtained from the BREEDIT gene discovery pipeline (Lorenzo *et al.,* 2023). These were generated by supertransforming immature embryos that are heterozygous for a Cas9-expressing T-DNA (EDITOR 1; Fig. 2b) with different SCRIPT constructs each containing 12 gRNAs (Fig. 2b) (Lorenzo *et al.,* 2023). We determined genotypes at target loci of primary transformants (T0 plants) by highly multiplex amplicon sequencing (HiPlex) followed by read-backed haplotyping to call edited alleles (Schaumont *et al.,* 2022). The T0 plants were homozygous, heterozygous, bi-allelic, or genetic mosaic at multiple targeted loci (Lorenzo *et al.,* 2023) (Fig. 3; Table S1).

We backcrossed T0 plants to wild-type B104 and the first backcross (BC1) generation plants were used as the starting material for haploid induction and ERD (Fig. 2a). Because the BC1 plants only contained heterozygous mutations, each gamete had a 50% probability of containing an edited allele at a given target locus. Hence, every gamete and resulting DH plant was expected to have a random combination of genetically unlinked edited loci. As no loci are heterozygous in DH0, the occurrence of a particular homozygous genotype of interest for *g* loci by self-crossing is increased from ${\left(\frac{1}{4}\right)}^{g}$ to ${\left(\frac{1}{2}\right)}^{g}$ (Fig. S1a, (Lübberstedt & Frei, 2012)). Self-crossing DH0 plants results in DH1 seeds with identical genotypes (Fig. 2a) which are suitable for replicated phenotyping.

We selected two T0 plants (T0_S4_001 and T0_S4_002) containing SCRIPT 4 with edits in ten of the twelve targeted genes (Fig. 3) (Lorenzo *et al.,* 2023). SCRIPT 4 targets seven class II CINCINNATA-TEOSINTE BRANCHED 1/CYCLOIDEA/PROLIFERATING CELL FACTOR (TCP) genes, and also *GROWTH REGULATING FACTOR4 (GRF4), GRF10* and *GRF17, BASIC PENTACYSTEINE 6 (BPC6)* and *PHD8,* a gene encoding a plant homeodomain-finger protein. These are all related to regulators of cell division, leaf shape, and leaf size determination. In the BREEDIT pipeline, populations edited with SCRIPT 4 showed strong final leaf 3 length (FLL3) and final leaf 3 width (FLW3) phenotypes. Although causative gene combinations are still unknown, the involvement of *GRF10* and various *TCP*s was hypothesized. All twelve SCRIPT 4 gRNAs were active and also in the T0 lines used here evidence of editing could be found in the sampled T0 leaves for all genes but *GRF17* and *PHD8* (Fig. 3) (Lorenzo *et al.,* 2023). Genotypes at each edited target locus ranged from homozygous for *TCP42* and bi-allelic edits for *GRF10* and *TCP9* to genetic mosaic for *TCP10.* We backcrossed T0 plants with wild-type B104 plants and 16 BC1 plants (eleven originating from T0_S4_001, five from T0_S4_002) were genotyped with HiPlex and scored for resistance to hygromycin (EDITOR T- DNA) and phosphinothricin (SCRIPT T-DNA). Genotyping confirmed the inheritance of edited alleles from T0 plants because Cas9-negative and/or SCRIPT-negative plants showed heterozygous edits with identical alleles also present in T0 (Fig. 3; Table S2). In addition, for BC1 plants that had both the SCRIPT 4 and EDITOR T-DNA, we observed transgenerational gene editing of the inherited wild-type B104 alleles (e.g. plant BC1_S4_006; Fig. 3; Table S2). We now also detected edits in *GRF17* and *PHD8* for which no mutations were observed in T0 (Tables **S1**, **S2**). We used these 16 BC1 plants and one wild-type B104 plant for haploid induction using RWS-GFP. In total, we obtained 173 plantlets after ERD, of which 165 were non-haploids. Of these, 148 were selected for genotyping (Figs 3; Table S3). As expected, the majority of DH0 plants were homozygous at all targeted loci (Figs 3; Table S3). As loci were mostly heterozygous in BC1, wild-type, and mutant alleles had an equal chance of being inherited for each genetically unlinked locus. For SCRIPT 4, only two target loci (*GRF4* and *PHD8*) are genetically linked (Lorenzo *et al.*, 2023). We indeed observed a variety of edited locus combinations in DH0 plants (Figs 3; Table S3). Twenty-four out of 148 DH0s showed additional transgenerational editing and were not used for future experiments. DH0 plants were transferred to the greenhouse for self-pollination to generate DH1 seeds. In total, 76 plants successfully yielded DH1 seeds. Of these DH lines, 34 contained unique homozygous edited combinations involving ten out of the twelve genes, with only edits in *GRF17* and *TCP3* lacking (Table S3). In conclusion, we confirmed that haploid induction and genome doubling can be performed using SCRIPT 4 edited plants, yielding a variety of homozygous DH lines with various combinations of edits.

### Phenotyping SCRIPT 4 DH1s unveils combinations of gene edits with increased leaf size

**Fig. 4:**
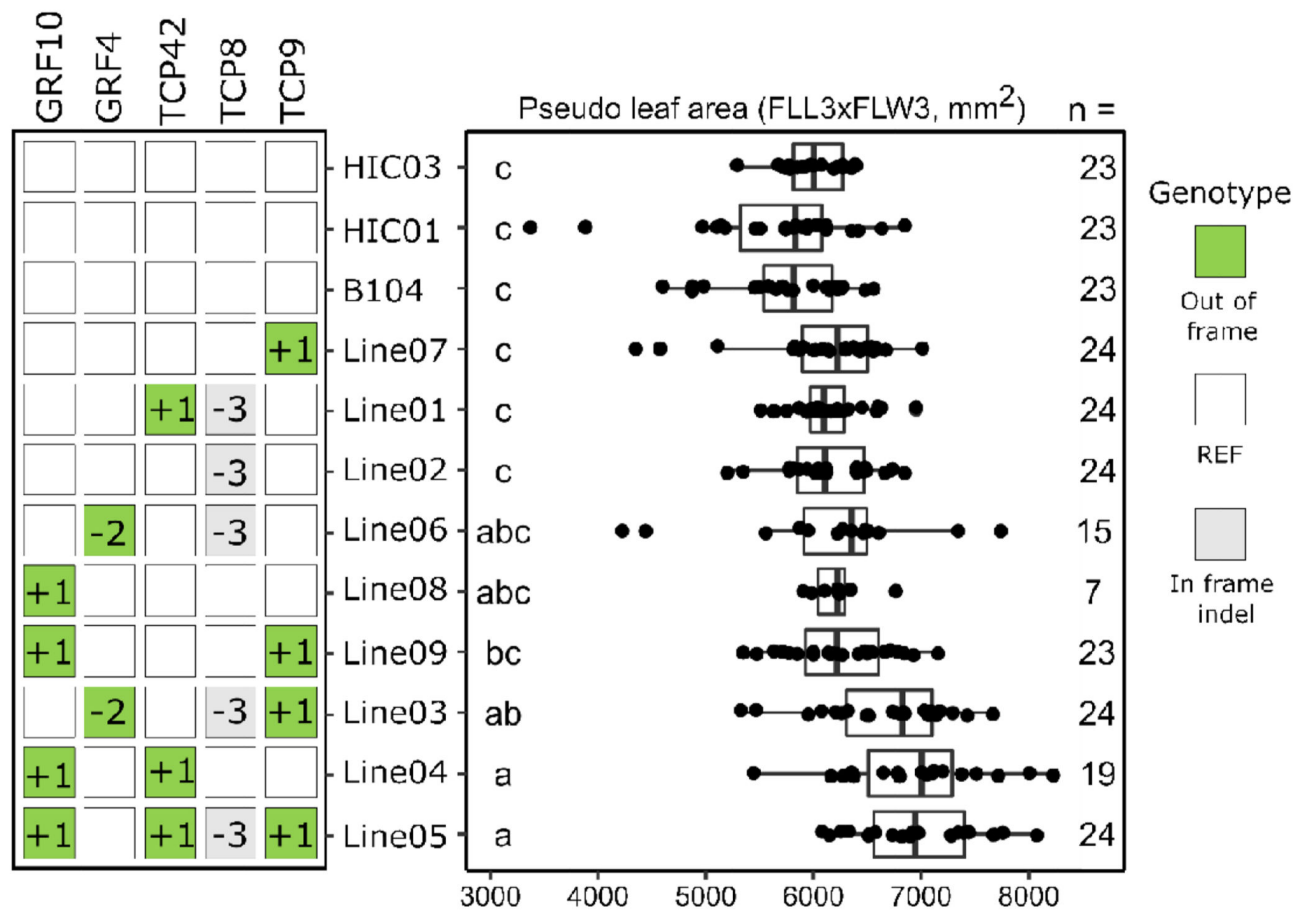
Phenotypic analysis of Zea mays (maize) SCRIPT 4 DH lines. Genotypes and corresponding phenotypes observed in DH1 SCRIPT 4 plants homozygous for various combinations of out-of-frame alleles (green squares) and in-frame mutated alleles (gray squares); the size of the indel (in bp) is indicated in the squares. White squares indicate that the wild-type reference (REF) allele was identified by genotyping. Each row represents an independent DH line. Boxplots with jittered data points on the right display measurements of pseudo leaf 3 area (PLA3) for edited plants compared with non-edited control plants (wild-type B104 and two wild-type doubled haploids (HIC01 and HIC03)). DH lines are sorted from lowest to highest mean PLA3. 24 to 29 seeds were sown for each DH line; n, number of germinated plants phenotyped. The compact letter display shows the result of the pairwise comparisons of the Wilcoxon rank sum test (significance level of 5% with Holm correction).

We selected nine DH lines derived from self-pollinated homozygous DH0 plants, with unique combinations that had a sufficient number of seeds available for phenotyping. We also included a wild-type B104 and two haploid-induced lines that only inherited wild-type alleles and went through the same procedures as the edited lines, as haploid-induced controls (HICs). We measured leaf phenotypes of DH1 plants as most target genes were selected to impact leaf growth. At V3 stage, final leaf 3 length (FLL3) and final leaf 3 width (FLW3) were measured and the pseudo leaf 3 area (PLA3, FLW3xFLL3) was calculated. Measurement of PLA is fast and non-destructive and the parameter correlates with total leaf area (Pearce *et al.*, 1975). Three edited DH lines displayed a significant increase in PLA3 compared with the controls (Figs 4): Line03 *(grf4;tcp8;tcp9,* on average 11.9% larger than HIC03), Line04 *(grf10;tcp42,* on average 15.3% larger), and Line05 *(grf10;tcp42;tcp8;tcp9,* on average 16.6% larger). Looking at FLL3, Line04 (*grf10;tcp42*) and Line05 (*grf10;tcp42;tcp8;tcp9*) were significantly different from the controls with an average increase of 8.9% and 8.8%, respectively compared with HIC01 (Fig. S4a). Further, for FLW3, only Line03 (*grf4;tcp8;tcp9*) showed a significant increase compared with the controls (on average 5.8% larger than HIC03) (Fig. S4b). With three different gene combinations having an increased PLA3, it is clear that the main driver for this increase is FLL3 for Line04 and Line05, while for Line03, FLW3 is clearly at the basis of the increased PLA3. From these results, the gene combination *grf10;tcp42* seemed to be the common denominator to have a positive effect on FLL3 (Fig. S4a). Because our method generates many different gene combinations, we were also able to include single mutants of the genes involved, a valuable asset to untangle the involved genes. Here, we observed no leaf phenotypes for *grf10* (Line08) or *tcp42* (with a *tcp8* in-frame allele, Line01) single out-of-frame mutants.

In conclusion, SCRIPT 4-edited DH plants were successfully used for replicated phenotyping, which revealed three stable edited gene combinations with an increased PLA3.

### Inter-SCRIPT crosses expand edited gene combinations even further

**Fig. 5:**
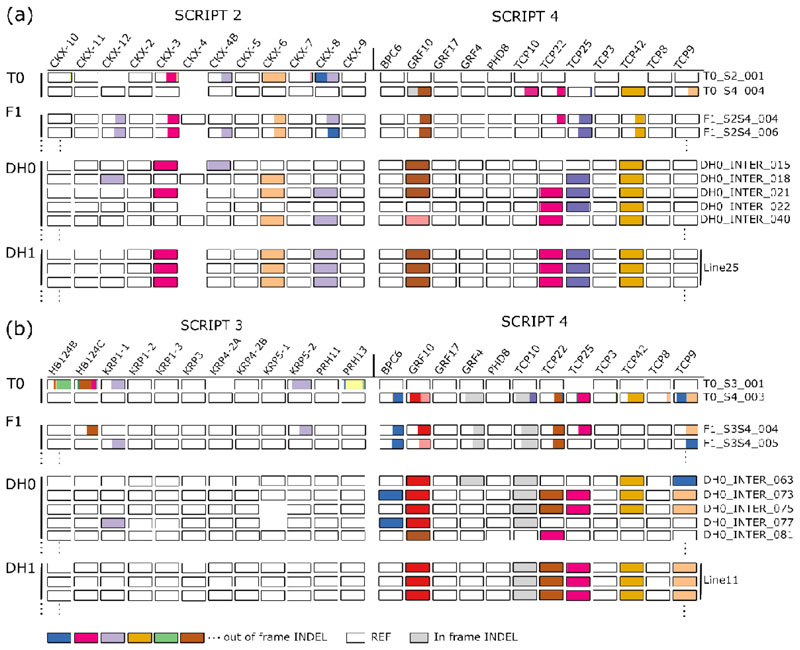
Combining haploid induction and multiplex gene editing in Zea mays (maize) with inter-script crosses. Experimentally obtained genotypes for a representative subset of plants of (**a**) S2xS4 and (**b**) S4xS3 are shown for each generation. Colored, horizontally stacked bars each indicate different mutant out-of-frame alleles per locus, white bars indicate wild-type (reference) alleles, light gray bars indicate in-frame mutant alleles and colored bar lengths are proportional to the fraction of sequence reads per locus containing the allele. Absent bars indicate missing data due to low-quality sequencing. Gene locus names are indicated above each column, as well as their respective SCRIPT construct.

To enlarge the possible combinations of gene-edited loci even further, we combined SCRIPT 4 with two other previously generated SCRIPTs (Lorenzo *et al.,* 2023). The different SCRIPT- targeted genes were distributed over the ten maize chromosomes with limited genetic linkage (Lorenzo *et al.,* 2023). SCRIPT 2 targets 12 members of the cytokinin oxidase (*CKX*) family and SCRIPT 3 targets 12 cell cycle- and drought-related genes. As the SCRIPTs target different genes, T0 plants for SCRIPT 4 are WT for SCRIPT 2- or SCRIPT 3-targeted genes and vice versa. T0 plants were selected with evidence for editing in 18 of the 36 targeted genes, again ranging from homozygous edited loci (e.g. for *CKX6*) to mostly WT, like for *TCP8* (Figs 5). T0 plants containing SCRIPT 2 were crossed with those containing SCRIPT 4 (inter-script cross, S2xS4) to yield an F1 ‘inter-script’ population with (heterozygous) edits in potentially up to 24 target loci. Similarly, SCRIPT 3 and SCRIPT 4 T0 plants were crossed to yield a second F1 inter-script population (S4xS3). In this experiment, we tested F1 plants for the absence of SCRIPT and/or EDITOR T-DNA to avoid transgenerational CRISPR/Cas9 editing further down the pipeline. Subsequently, we selected six F1 plants from each inter-script F1 population that lacked the Cas9-containing EDITOR T-DNA.

HiPlex amplicon sequencing confirmed that plants contained heterozygous mutations or wild-type alleles at the targeted loci (Figs 5, Table S2). Twelve F1 plants were pollinated with RWS-GFP pollen and resulting haploid embryos were subjected to the ERD procedure. 138 haploid embryos were identified and treated with colchicine (HIR = 15.3%), and 123 survived treatment (survival = 89.1%). After flow cytometry analysis, 87 DH0 plants were transferred to the greenhouse for self-pollination.

Similarly, as with SCRIPT 4 alone, 73 out of 87 inter-script DH0 plants contained a variety of combinations of homozygous edits and wild-type alleles, but now spanning two SCRIPTs (Figs 5, Table S3). Fourteen out of 87 DH0 plants did not exclusively contain homozygous mutations; several heterozygous and bi-allelic mutations were observed. We attribute this to either accidental self-pollination of F1 plants or misidentification of GFP absence. Hence these DH0s were removed from further phenotypic analysis. Confirmed DH0 plants were grown to maturity in the greenhouse and plants that simultaneously formed silks and pollen at the correct interval were self-crossed. In total, we obtained DH1 progeny for 44 independent DH0 plants, including 25 unique homozygous edited combinations. To summarize, we were able to create homozygous lines from inter-script F1 plants using haploid induction and ERD. We obtained lines with up to seven homozygous out-of-frame edits suitable to be used in replicated phenotyping.

### A genetically stable ckx3;6;8 tcp22;25;42 grf10 septuple mutant reproducibly shows increased leaf size

Similar to SCRIPT 4, we used a selection of inter-script DH lines for replicated phenotyping. To screen many lines simultaneously, 6 to 10 replicates per DH line were phenotyped for PLA3, demonstrating that several lines indeed showed a significantly increased PLA3 (Line11, Line20, and Line25, Fig. S5). For FLL3, no lines were found to be significantly different compared with the controls (Fig. S6a), and for FLW3, two lines showed a significant increase (Line11 and Line25, Fig. S6b). To confirm the stability of the genotypes, we sequenced the DH1 plants of the three lines with the best PLA3 performance, and four HIC individuals (Figs 5, Table S4). Since all sequenced DH1 individuals showed homozygous genotypes, we could conclude that DH lines originating from homozygous DH0 plants are genetically stable.

**Fig. 6:**
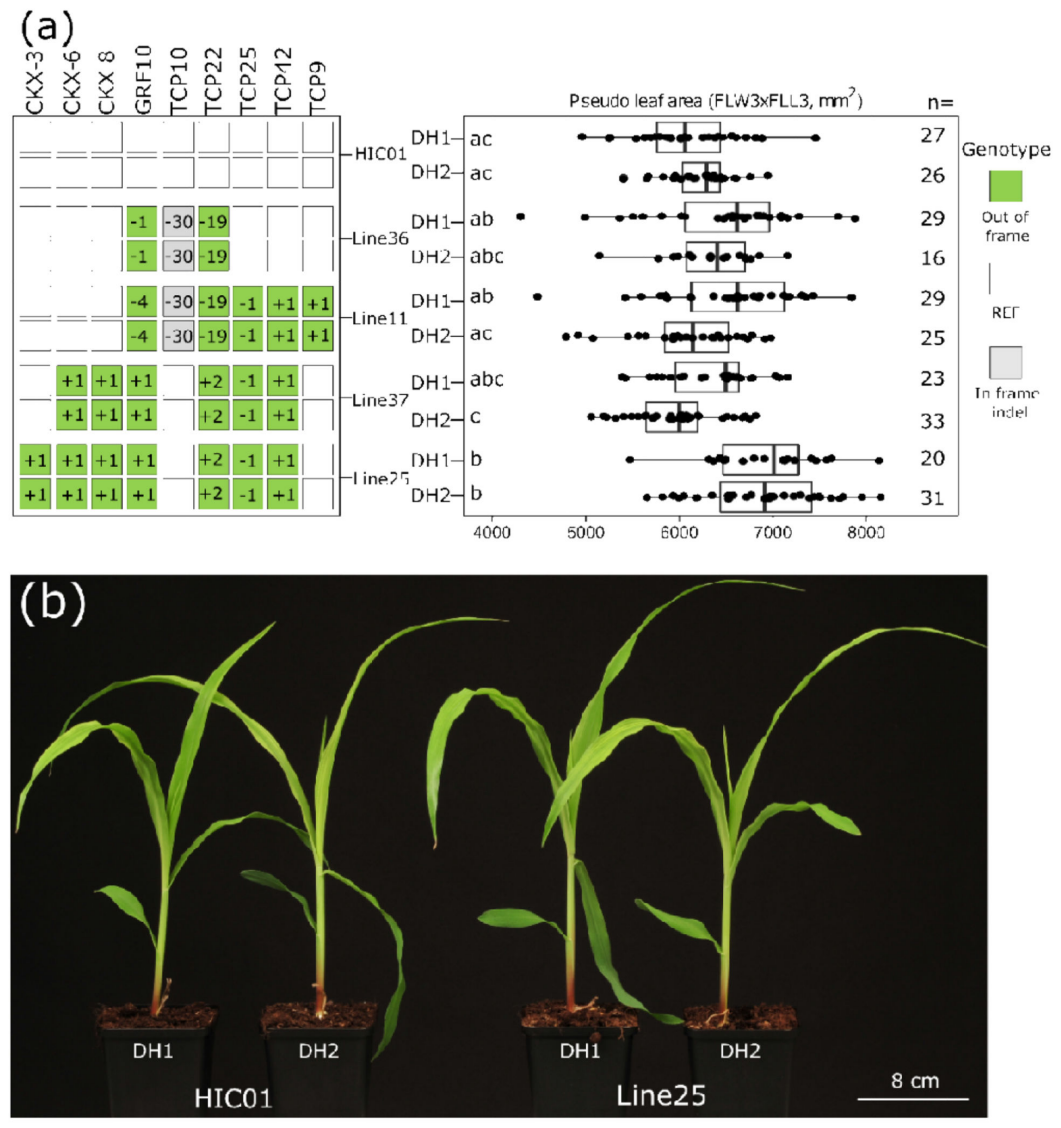
Phenotypic analysis of Zea mays (maize) DH1 and DH2 plants. (**a**) Observed genotypes and corresponding phenotypes in DH1 and DH2 for four different homozygous edited genotypes and the non-edited control HIC01. Out-of-frame mutated alleles (green squares), in-frame mutated alleles (gray squares) and reference alleles (white squares), the size of the indel (in bp) is indicated in the squares. On the left, each row represents the genotype of a line (DH1 or DH2 generation), on the right, corresponding boxplots with jittered data points display measurements of pseudo leaf 3 area (PLA3). 24 to 35 seeds of each line were sown; n, number of plants phenotyped. The compact letter display shows the result of the pairwise comparisons of the Wilcoxon rank sum test (significance level of 5% with Holm correction). (**b**) Representative seedlings at V3 stage for HIC01 and the septuple mutant Line25, one plant of each generation (DH1 and DH2).

Using a power analysis for PLA3, we estimate that at least 12 genetically identical plants are needed to significantly attribute a 10% difference in PLA3 with 80% statistical power (Fig. S7). We selected four genotypes based on the preliminary phenotypic screen for a repeated experiment, now with more individuals per line. In general, DH0 B104 plants growing in the greenhouse appeared to be smaller, often lacked proper ear and tassel development, or had a desynchronized ear and pollen maturity, leading to a lower reproduction rate and lower seed set compared with plants that did not undergo colchicine treatment. We hypothesized that this poor DH0 performance might also impact its DH1 progeny in phenotyping experiments, adding variability. Hence, for comparison, we also included corresponding DH2 plants, generated by self-crossing a DH1 plant (Fig. 6a). Four lines were selected alongside the HIC01 control, including *ckx3;6;8 tcp22;25;42 grf10* (Line25) and *tcp9;10;22;25;42 grf10*(Line11), which performed well in the preliminary phenotypic screen of the inter-script DH1s (Figs S5, S6b), and also two new genotypes, *tcp10;22 grf10* (Line36) and *ckx6;8 tcp22;25;42 grf10* (Line37); Line37 only differed from Line25 by the absence of an edit in *CKX3*. For each of the four selected genotypes and the HIC, a representative DH1 plant was genotyped, again confirming genetic stability (Tables **S3**, **S4**). For all four lines and the HIC01, there was no significant difference in PLA3 or FLL3 between the respective DH1 and DH2 lines (Figs 6, S8a). For FLW3, there was however a significant difference between DH1 and DH2 for Line11 and Line37 (Fig. S8b). For both DH1 and DH2, the septuple mutant *ckx3;6;8 tcp22;25;42 grf10* (Line25) had a strongly increased PLA3 compared with HIC01, 13.1% higher for DH1 and 10.8% higher for DH2. Interestingly, Line37, which only differs from Line25 in lacking the *ckx3* mutation, does not have a significantly increased PLA3, FLL3, or FLW3 compared with the control (Figs 6, S8). This suggests a critical role for *ckx3* in the expression of this phenotype. The expression profile of *CKX3* in relevant organs is most similar to *CKX6* in publicly available maize gene expression data (Fig. S9) (Stelpflug *et al.,* 2016). Therefore, the simultaneous mutation of *CKX3* and *CKX6* in Line25 might explain the increase in PLA3 as compared to Line37 in which only *CKX6* is edited.

In conclusion, DH1 generation plants can already be used for screening PLA3, and in only three generations, we established a collection of higher-order mutants, of which a septuple *ckx3;6;8 tcp22;25;42 grf10* mutant reproducibly showed an enlarged PLA3.

## Discussion

To engineer a complex quantitative trait such as leaf growth, combining alleles affecting distinct processes, such as cell proliferation and expansion, is required (Vanhaeren *et al.,* 2014). However, analysis of gene function, dissection of molecular networks, and genetic improvement using loss-of-function approaches are hampered by the prevalence of functional redundancy of duplicated genes in crops. Maize has also undergone several genome duplication events during its evolution (Schnable *et al.*, 2009). When multiple copies of a gene are present with identical or highly overlapping expression and function, higher-order mutants are needed to observe phenotypes.While efforts are made to predict gene redundancy within gene families based on parameters such as gene expression (Cusack *et al.*, 2021), experimental testing of combinatorial loss-of-function mutants is often desired to study gene families (Hall & Bleecker, 2003).

CRISPR/Cas9 multiplexing allows efficient and simultaneous editing of multiple genes in maize (Xing *et al.,* 2014). The subsequent generation of stable plants with desired combinations of homozygous edits (or wild-type alleles) at targeted loci for further detailed characterization may require several generations of genetic crosses combined with genotypic selection. Although this can be sped up by self-crossing, it is best practice to backcross maize primary transformants to the corresponding wild-type background to reduce any potential somaclonal variation from the transformation process (Bregitzer *et al.*, 2008). A particular homozygous edit is then encountered at ${\left(\frac{1}{4}\right)}^{n}$ chance by a self-cross following this backcross. Because numbers become impractical starting from 5 multiplex edited loci, there is a need for a simple, effective strategy to create fully homozygous edited plants when multiplexing.

The effort to obtain a particular higher-order combination is greatly reduced by our GEDH strategy presented here, combining CRISPR/Cas9-mediated multiplex gene editing with *in vivo* haploid induction and efficient *in vitro* generation of doubled haploid plants using embryo rescue doubling. First, multiplex edited maize plants are backcrossed to wild-typeB104, after which plants that are either heterozygous or wild-type at each locus are pollinated with HI pollen. Then, selected haploid embryos are treated with colchicine to double their chromosomes, after which they are germinated *in vitro*. Non-haploid plants are then continued to the greenhouse and self-pollinated to produce identical homozygous DH1 progeny. With a high HIR, easy haploid identification, and highly efficient haploid doubling, our GEDH approach can be conducted in most labs with basic tissue culture experience and appropriate infrastructure. We found that the main factors limiting our approach were the variability in the success of self-crossing of the DH0 plants, and the greenhouse space needed to grow them. We limited the use of space by selecting only non-haploids based on flow cytometry, but as on average only 7.8% was found haploid, this step might be unnecessary, especially if space is not an issue.

Several alternative HI lines with a high HIR are available for maize (Kalinowska *et al.,* 2019). Moreover, the major genes underlying the HI phenotype, *mtl* and *dmp* have been identified (Gilles *et al.,* 2017; Kelliher *et al.,* 2017; Liu *et al.,* 2017; Zhong *et al.,* 2019). Creating a HI line in the B104 background, which is often used by the research community, would have an advantage over RWS, because it would have a similar flowering time as the multiplex edited lines, facilitating the planning of experiments. In the currently used RWS-GFP HI line, GFP is driven by the 35S promoter that is expressed both in the embryo and endosperm (Yu & Birchler, 2016). Making use of an embryo-specific promoter to drive a fluorescent marker could improve accurate haploid embryo identification (Dong *et al.,* 2018). As the maternal HI system based on *mtl* has been translated into other grasses such as wheat (C. Liu *et al.*, 2020; Sun *et al.*, 2022) and rice (Yao *et al.*, 2018), our GEDH strategy can also be applied to other crops in the future. For hexaploid wheat, loss-of-function mutations in all six homoeoalleles are often needed to obtain desirable phenotypes (Wang *et al.*, 2014). Another application (HI-Edit) that combines the doubled haploid technology and gene editing has recently been reported. HI-Edit is distinct from our approach and uses paternal genome elimination to transiently deliver CRISPR/Cas9 to recalcitrant, elite maize lines (Kelliher *et al.*, 2019; Wang *et al.*, 2019) or wheat (Kelliher *et al.*, 2019; Budhagatapalli *et al.*, 2020) by pollination and bypasses tissue culture.

Even in highly controlled growth conditions, genetically identical plants are desired as biological replicates to analyze quantitative traits such as leaf growth and to reliably associate phenotypes with genotypes (Lorenzo *et al.,* 2023). For the same reason, the Nested Association Mapping and similar homozygous maize lines are often used to study complex quantitative traits (Yu *et al.,* 2008), often obtained after haploid doubling (Mayer *et al.,* 2022). Here, we show that from one multiplex gene editing transformation event, an array of combinatorial homozygous mutants can be obtained in just three generations. The resulting DH1 seeds can immediately be used for phenotypic analysis and have immortal genotypes when self-crossed. While the genotype of these plants is completely stable, one has to consider epigenetic changes during DH production, which changes the chromatin environment and might affect gene expression. In *Arabidopsis,* it was recently found that haploidization and colchicine-induced genome doubling produced differentially methylated DNA regions (Piskorz *et al.,* 2023). While expression of protein-coding genes was only weakly affected and no phenotypic differences were found, it is advised to consider epigenetic changes after DH production and confirm phenotypes with independent DH lines.

Several experimental design strategies have been proposed for multiplex CRISPR screens in plants using combinatorial gRNA libraries. These strategies aim to maximize coverage of relevant genetic interactions while minimizing the number of plants (Van Huffel *et al.,* 2022). Our use of GEDH can be considered as variations of these strategies in which first higher- order mutants are generated and subsequently deconvoluted to identify causative genotypes for the phenotypes of interest (Fig. S10). Even with the advantage of using DH compared to self-crossing, obtaining and maintaining all possible 4096 DH lines covering all combinations of 12 targets is challenging. However, partial screens covering a subset of combinations, followed by a complete screen of all combinations of remaining targets can be achieved using iterative rounds of GEDH (Fig. S10). Another potential goal of using GEDH is the targeted generation and identification of a specific, predefined homozygous higher- order mutant line (Fig. S10). For example, one may want to obtain a line with stacked homozygous edits. After self-crossing BC1 and screening 1000 progeny, there is a 22% chance of obtaining a specific line homozygous for six target genes. By contrast, only 146 DH lines are required to obtain the sextuple mutant with a probability of 90% (Fig. S1b, (Lübberstedt & Frei, 2012)). If the number of targets is higher (*e.g.* 1000 DH lines for nine targets), different selection strategies can be used. These include self-crossing preceding the haploid doubling (Bonnett *et al.*, 2005) and a strategy in which DH plants are selected with matching genotypes, crossed, and the progeny used as founders in another round of GEDH to obtain the higher-order edited combinations (Ishii & Yonezawa, 2007).

Here, we were able to produce several homozygous edited maize lines that showed an increase in the pseudo leaf area of the third leaf at V3. Also, mature-stage phenotypes have been observed in plants with altered seedling leaf area (Nelissen *et al.*, 2015; Sun *et al.*, 2017), and it will be interesting to investigate if these lines also show an increase in biomass and/or seed yield in the field. Among several edited gene combinations, a double mutant *grf10;tcp42* showed a 15.3% increase in 3^rd^ leaf area. Further, a septuple mutant including *grf10;tcp42* alleles also displayed an increase in 3^rd^ leaf area of more than 10% (10.8-13.1%). This demonstrates the combined power of the BREEDIT strategy (Lorenzo *et al.,* 2023), which can rapidly scan the gene space, and GEDH, which can fix candidate gene combinations and produce stable homozygous lines for replicated phenotyping.

## Supplementary Material

Supplementary Figures

Supplementary Tables

## Data Availability

The data that supports the findings of this study are available in the supplementary material of this article. Sequencing data have been deposited on NCBI under BioProject PRJNA815957.
