# Supplementary material for: Combining multiplex gene editing and doubled haploid technology in maize
Source: New Phytol. Author manuscript; Available in PMC 2024 Feb 1. (PMC7614789; doi:10.1111/nph.19021)
Supplement: Supplementary Figures [file EMS180725-supplement-Supplementary_Figures.pdf]

***New Phytologist* Supporting Information**

Article title: Combining multiplex gene editing and doubled haploid technology in maize

Authors: Lennert Impens, Christian D. Lorenzo, Wout Vandeputte, Pieter Wytynck, Kevin Debray, Jari Haeghebaert, Denia Herwegh, Thomas B. Jacobs, Tom Ruttink, Hilde Nelissen, Dirk Inzé and Laurens Pauwels

The following Supporting Information is available for this article:

**Fig. S1. Comparison of self-pollination with haploid doubling.**

**Fig. S2. Maize (*Zea mays*) cob and isolated embryos after a cross of B104 (female) and RWS-GFP (male).**

**Fig. S3. Examples of three different flow cytometry outcomes after *Zea mays* (maize) haploid doubling.**

**Fig. S4. Phenotypic analysis of *Zea mays* (maize) SCRIPT 4 DH1 lines.**

**Fig. S5. Phenotypic screen of PLA3 in *Zea mays* (maize) inter-script DH1 lines.**

**Fig. S6. Phenotypic screen of FLL3 and FLW3 in *Zea mays* (maize) inter-script DH1 lines.**

**Fig. S7. Power analysis for the use of PLA3 in replicated phenotyping of *Zea mays* (maize).**

**Fig. S8. Phenotypic analysis of *Zea mays* (maize) DH1 and DH2 plants.**

**Fig. S9. Expression of *Zea mays* (maize) *CKX* genes targeted using SCRIPT 2.**

**Fig. S10. Overview of possible GEDH strategies and goals.**

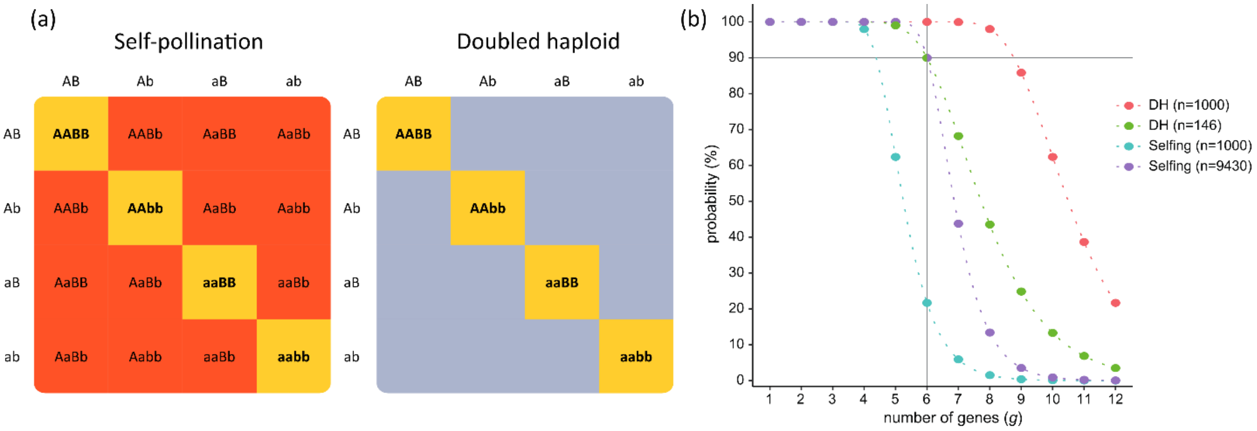


**Fig. S1 Comparison of self-pollination with haploid doubling.** (**a**) Genotype distribution after self-pollination compared with haploid doubling. On the left, the Punnett square shows all possible genotypes for progeny after self-pollination of a heterozygous parent plant (two independently segregating loci as an example). A particular homozygous genotype (e.g. AABB) occurs with a frequency of $\left(\frac{1}{4}\right)^{n}$ for n independently segregating loci. On the right, the Punnett square displays the genotype distribution for progeny after using a heterozygous plant as a parent for haploid induction and subsequent ERD (two loci as an example). A particular homozygous genotype (e.g. AABB) now occurs at a frequency of $\left(\frac{1}{2}\right)^{n}$ for n independently segregating loci. (**b**) Probability to obtain at least one individual with a favorable genotype, homozygous for 1–12 target genes. Different scenarios are plotted (self-crossing or doubled haploids, n = population size). Population sizes were chosen based on a realistic number of plants that we can grow in our facility or produce with GEDH. Currently we use an area of 6.6 $m^2$ to grow 1000 plants to at least V3. For six genes with a probability of 90%, one would need at least 146 DH plants or 9430 self-crossed progeny to find a particular genotype; 146 is approximately the amount of plants we produced here with one round of GEDH.

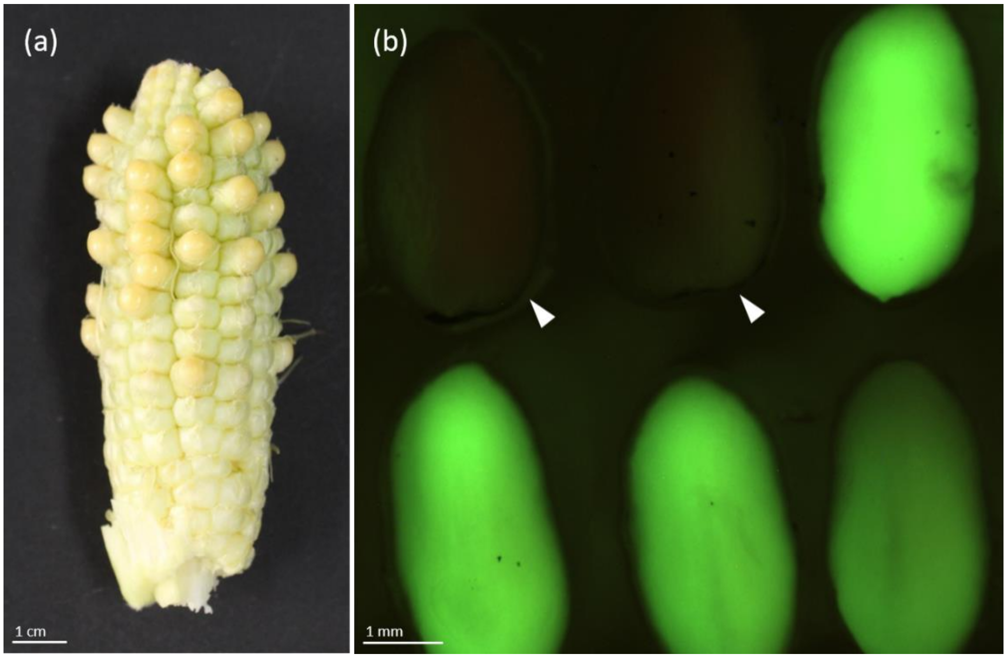


**Fig. S2 Maize (*Zea mays*) cob and isolated embryos after a cross of B104 (female) and RWS-GFP (male).** (**a**) Cob harvested 14 days after an *in vivo* haploid induction cross. At this stage, embryos were isolated and haploids separated from diploids. (**b**) Haploid maize embryos (indicated by white arrowheads) were scored by the absence of GFP expression (RWS-GFP genome elimination). Diploids show GFP expression with the RWS-GFP genome still present.

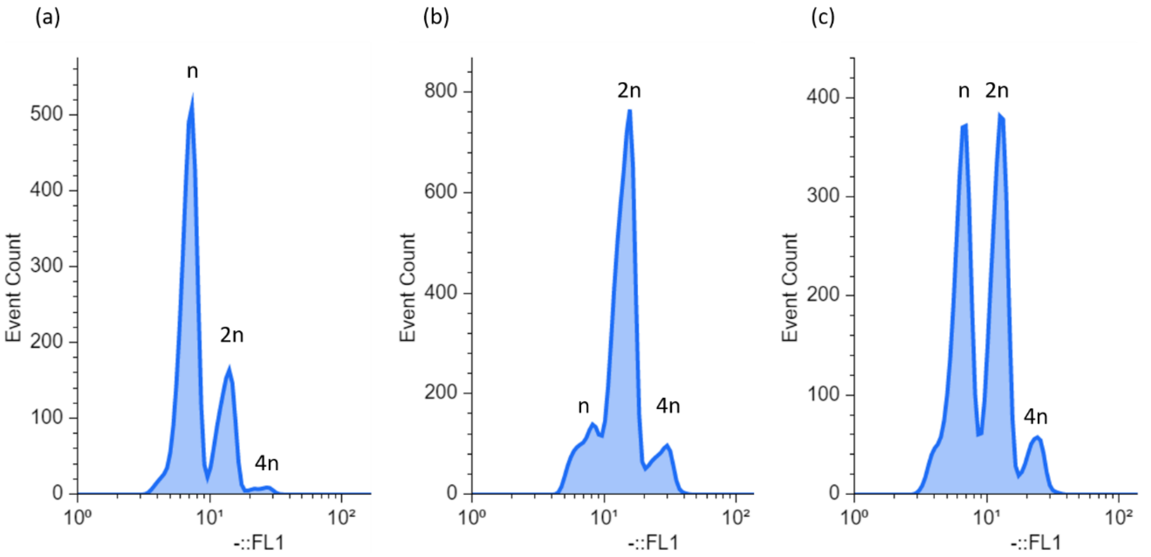


| Ploidy score: | Haploid | Diploid | Mixoploid |
|---|---|---|---|
| Plant number: | Not continued | DH0_S4_021 | DH0_S4_012 |

**Fig. S3 Examples of three different flow cytometry outcomes after *Zea mays* (maize) haploid doubling.** Graphs represent DNA content measured by CyFlow®ML cytometer (Partec), x-axes represent DNA content (fluorescent channel 1, FL1), y-axis shows event count. Example histogram of a (**a**) haploid plant, (**b**) diploid plant, and (**c**) mixoploid plant. Peaks are labeled with their respective ploidy number. Below the graphs, ploidy scores and the plant numbers (as seen in Table S3) are shown.

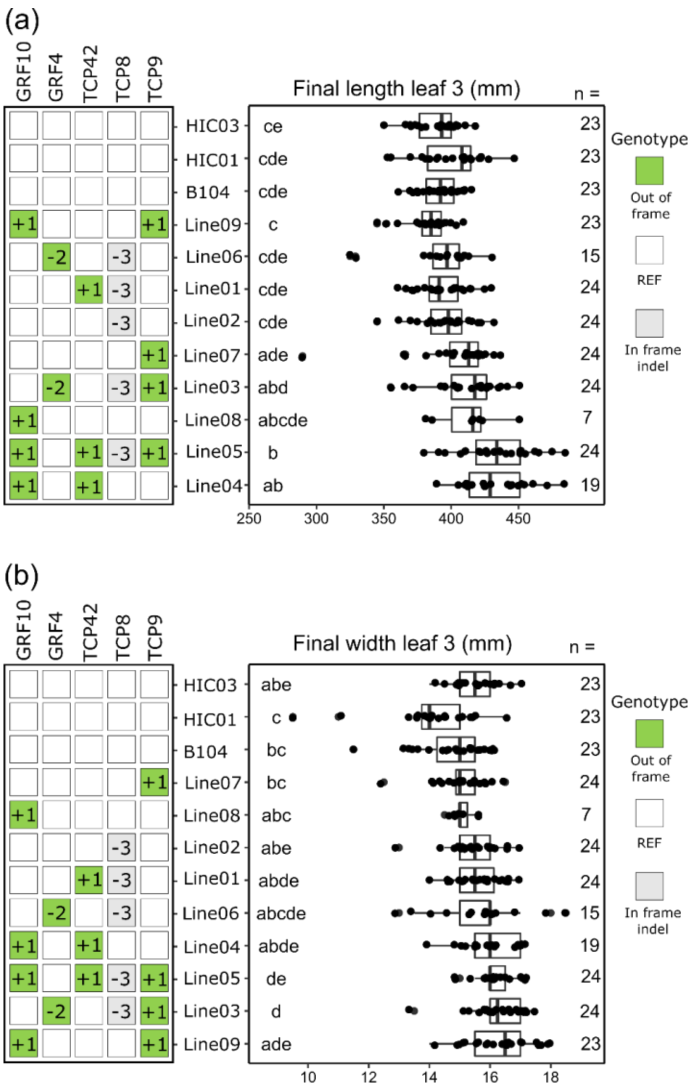


**Fig. S4 Phenotypic analysis of *Zea mays* (maize) SCRIPT 4 DH lines.** Genotypes and corresponding phenotypes observed in DH SCRIPT 4 lines homozygous for various combinations of out-of-frame alleles (green squares) and in-frame mutated alleles (gray squares); the size of the indel (in bp) is indicated in the squares. White squares indicate that the wild-type reference (REF) allele was identified by genotyping. Each row represents an independent DH line. Boxplots with jittered data points on the right display measurements of (**a**) final leaf 3 length (FLL3) and (**b**) final leaf width (FLW3) for edited plants compared with non-edited control plants (wild-type B104 and two wild-type doubled haploids (HIC01 and HIC03)). DH lines are sorted from lowest to highest mean FLL3 (**a**) or FLW3 (**b**). 24 to 29 seeds were sown for each DH line; n, number of germinated plants phenotyped. The compact letter display shows the result of the pairwise comparisons of the Wilcoxon rank sum test (significance level of 5% with Holm correction).

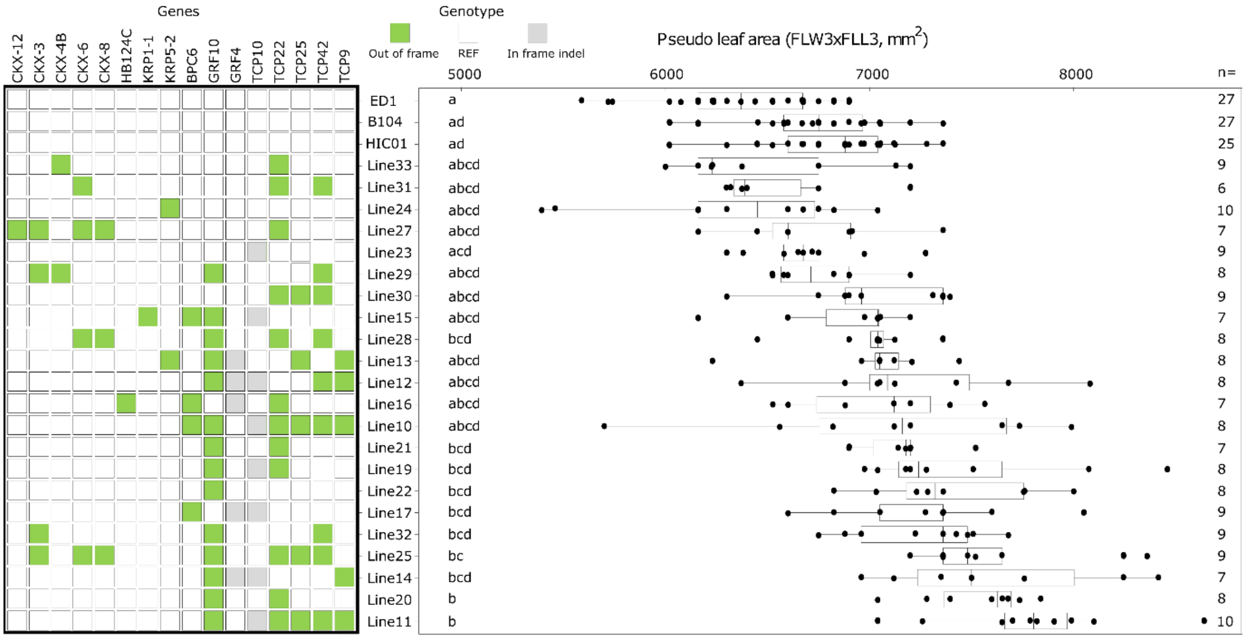


**Fig. S5 Phenotypic screen of PLA3 in *Zea mays* (maize) inter-script DH lines.** Genotypes and corresponding phenotypes observed in DH1 inter-script plants homozygous for various combinations of out-of-frame alleles (green squares) and in-frame mutated alleles (gray squares). White squares indicate that the wild-type reference (REF) allele was identified by genotyping. Each row represents an independent DH line. Boxplots with jittered data points on the right display measurements of pseudo leaf 3 area (PLA3) for edited plants compared with non-edited control plants (wild-type B104, EDITOR 1 without SCRIPT (ED1) and a wild-type doubled haploid (HIC01)). DH lines are sorted from lowest to highest mean PLA3. Eight to twelve DH1 seeds were sown for each DH line and 30 seeds for three control lines; n, number of germinated plants phenotyped. The compact letter display shows the result of the pairwise comparisons of the Wilcoxon rank sum test (significance level of 5% with Holm correction). Absent squares for *TCP42* indicate missing data due to low-quality amplicons. The FLL3 and FLW3 components of PLA3 are plotted individually in Fig. **S6**.

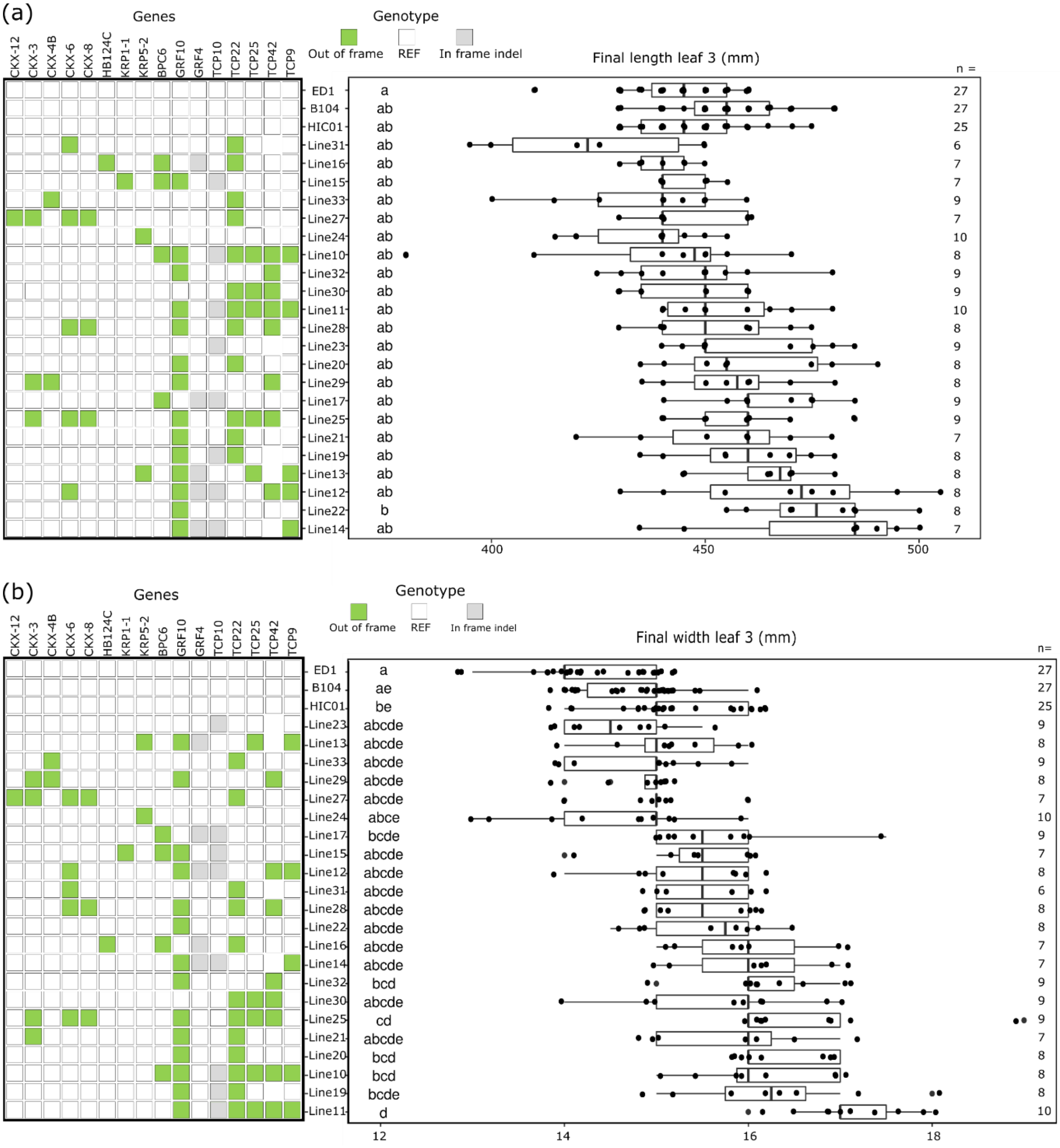


**Fig. S6 Phenotypic screen of FLL3 and FLW3 in *Zea mays* (maize) inter-script DH lines.** Genotypes and corresponding phenotypes observed in DH1 inter-script plants homozygous for various combinations of out-of-frame alleles (green squares) and in-frame mutated alleles (grey squares). Each row represents an independent DH line. Boxplots with jittered data points on the right display measurements of (**a**) final leaf 3 length (FLL3) and (**b**) final leaf width (FLW3) for edited plants compared to non-edited control plants (wild-type B104, EDITOR 1 without SCRIPT (ED1)

and a wild-type doubled haploid (HIC01)). DH lines are sorted from lowest to highest mean FLL3 (**a**) or FLW3 (**b**). Eight to twelve DH1 seeds were sown for each DH line and 30 seeds for three control lines; n, number of germinated plants phenotyped. The compact letter display shows the result of the pairwise comparisons of the Wilcoxon rank sum test (significance level of 5% with Holm correction). Absent squares for *TCP42* indicate missing data due to low-quality amplicons.

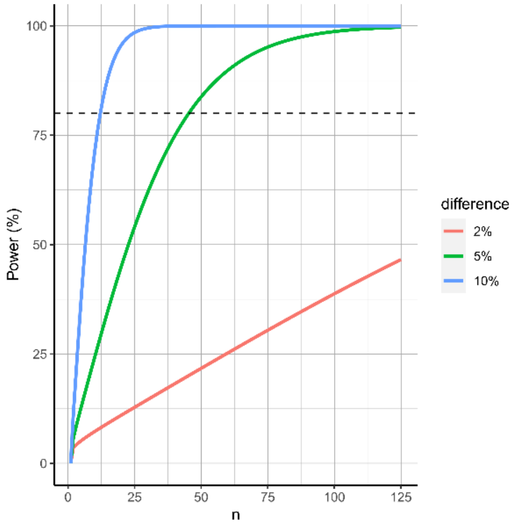


**Fig. S7 Power analysis for the use of PLA3 in replicated phenotyping of *Zea mays* (maize).** X-axis shows the number of plants (n) required to show a 2%, 5%, or 10% significant difference in PLA3 with a power of 80% (dashed horizontal line). Based on B104 wild-type variation seen in PLA3 measurements over the phenotypic experiments in this manuscript, assumption of normality and using a t-test.

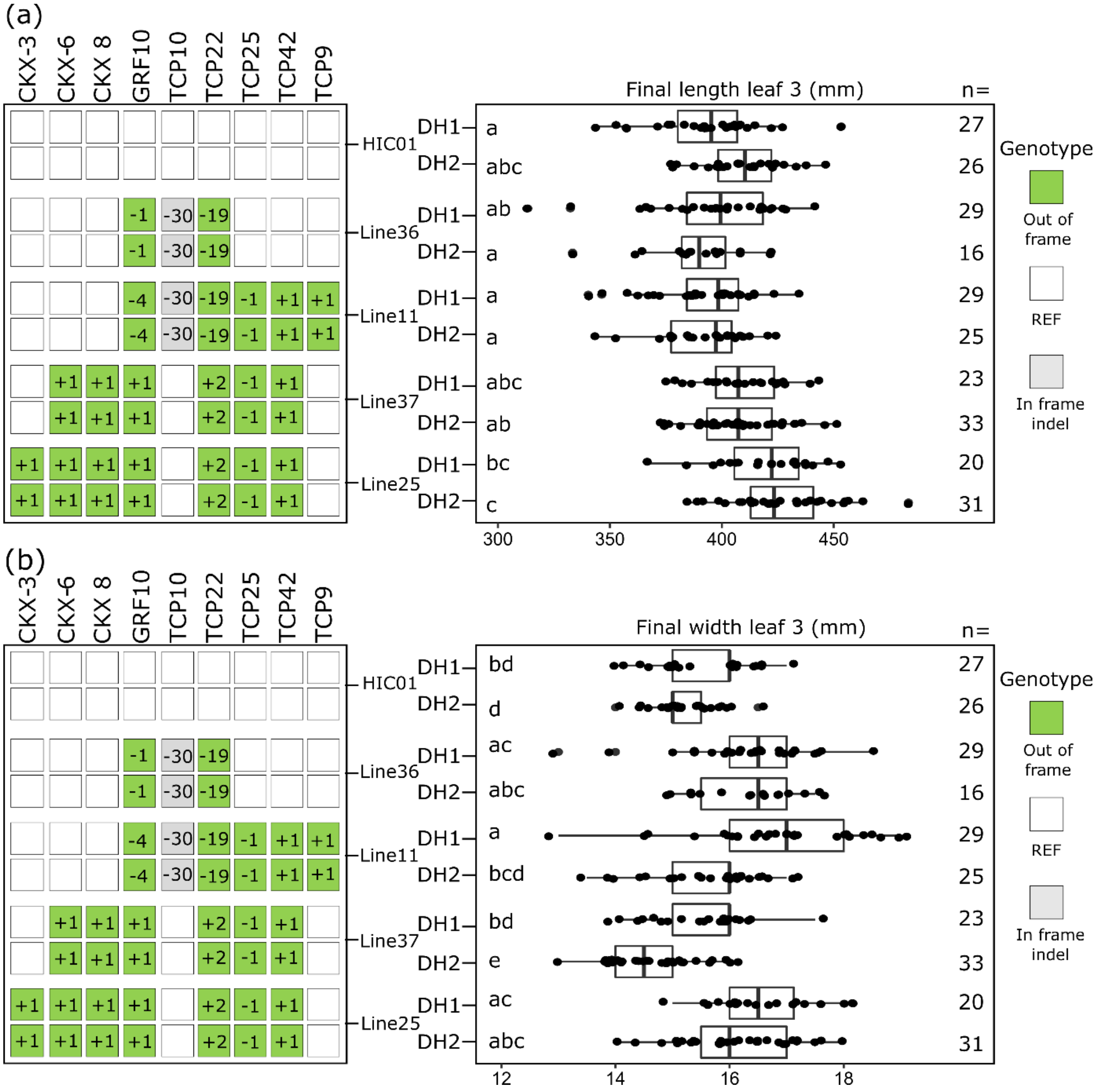


**Fig. S8 Fig. S8. Phenotypic analysis of *Zea mays* (maize) DH1 and DH2 plants.** Genotypes and corresponding phenotypes observed in DH1 and DH2 generations for four different homozygous edited lines and non-edited control lines (HIC01). Out-of-frame mutated alleles (green squares), in-frame mutated alleles (grey squares) and reference alleles (white squares), the size of the indel (in bp) is indicated in the squares. On the left, each row represents the genotype of a line (DH1 or DH2), on the right, corresponding boxplots with jittered data points display measurements of (**a**) final leaf 3 length (FLL3) and (**b**) final leaf width (FLW3). 24-35 seeds of each line were sown; n, number of germinated plants phenotyped. The compact letter display shows the result of the pairwise comparisons of the Wilcoxon rank sum test (significance level of 5% with Holm correction).

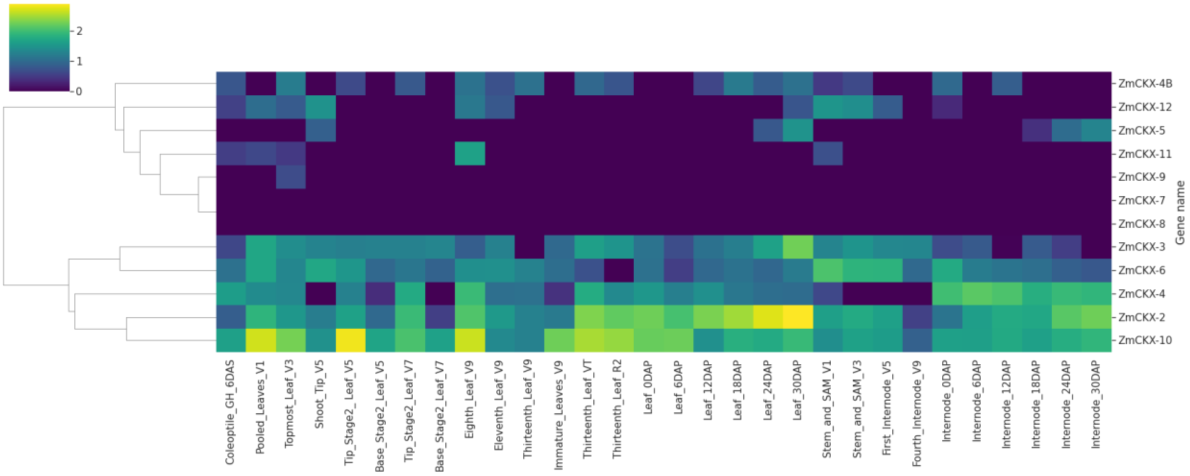


**Fig. S9 Expression of *Zea mays* (maize) *CKX* genes targeted using SCRIPT 2**. For each *CKX* gene, log10 transformed FPKM values for internode and the organ groups leaf, shoot apical meristem (SAM) and young stem from the Maize Gene Expression Atlas (Stelpflug *et al.*, 2016) are plotted as a heat map. The heatmap and the hierarchical clustering of the genes presented here was created using the clustermap function of the seaborn package (Hunter, 2007; Waskom, 2021). DAS, days after sowing; Vn, vegetative stage corresponding to the number of emerged leaves; VT, vegetative tasseling; DAP, days after pollination; R2, reproductive 2 stage. All samples are from field-grown plants, except coleoptile (GH, greenhouse).

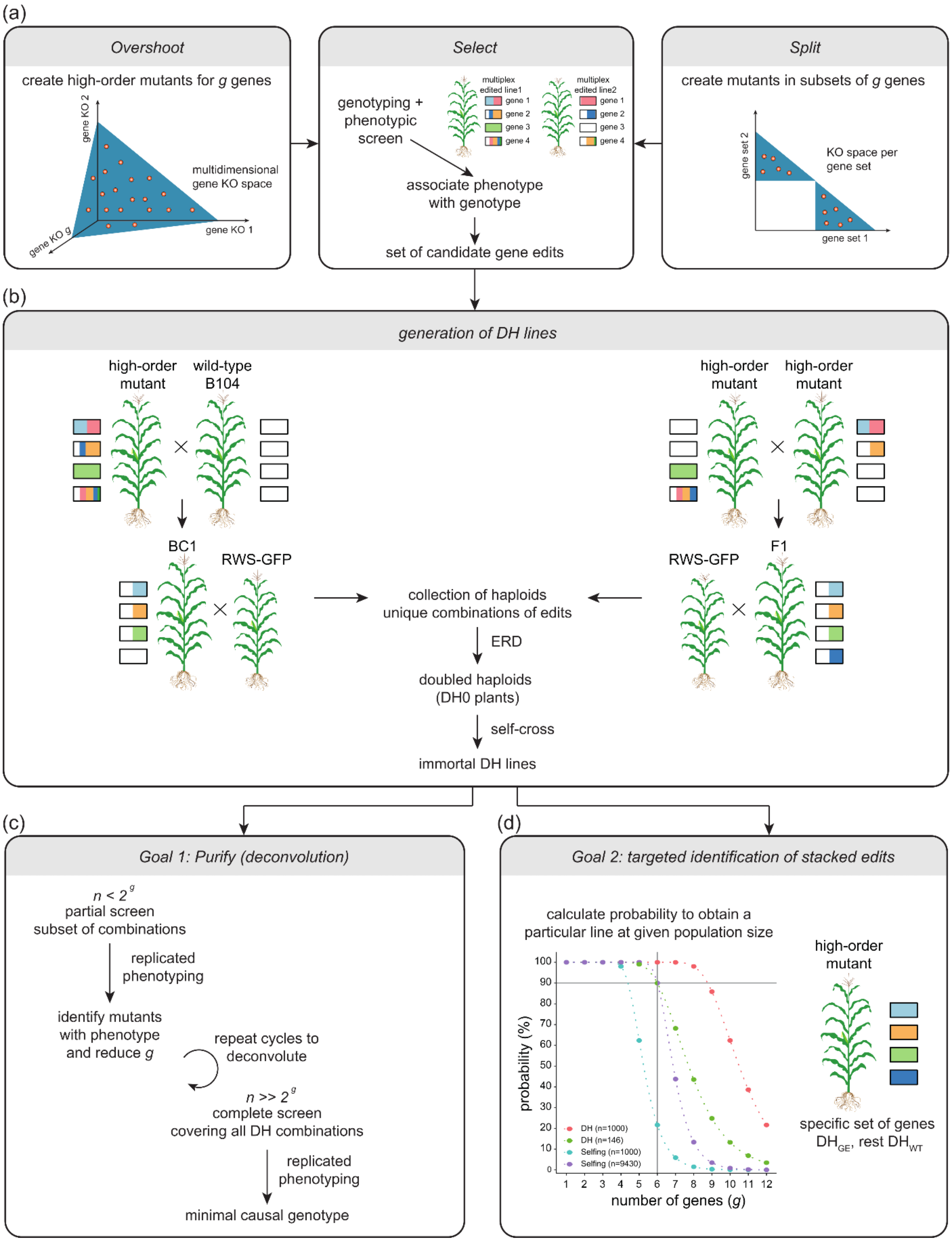


**Fig. S10 Overview of possible GEDH strategies and goals**. (**a**) Populations of mutants are made covering the target gene (*g*) space either with one SCRIPT with many targets (*Overshoot*) or

multiple SCRIPTS with subsets of targets (*Split*). See (Van Huffel *et al.*, 2022) for detailed explanation of the efficiencies of those strategies. After genotyping and phenotyping the mutant collection, interesting phenotypes are associated with gene edits (*Select*). This approach has been performed in Lorenzo *et al*., 2022. Based on the association data, candidate gene sets are defined. (**b**) High-order mutants carrying those mutations are selected as starting point for GEDH. For simplicity, four GE target loci are shown to represent a genotype, stacked colored bars represent different edited alleles, and white bars indicate reference alleles, bar lengths are proportional to the allele frequencies. After crossing these mutants with wild-type B104 (BC1), or with each other (F1; in the BREEDIT strategy called "interscript cross"), heterozygous progeny is used for haploid induction and the generation of doubled haploids via embryo rescue doubling (ERD). Self-crossing those doubled haploids results in a collection of immortal DH lines, differing in edit combinations as identified by HiPlex genotyping. (**c**) A first possible goal of GEDH is to identify a minimal causal genotype based on replicated phenotyping of various DH lines (*Purify* for gene deconvolution). If the number of target genes (*g*) is high, the number of DH lines feasible to generate is far smaller than all possible combinations ($n < 2^g$). Then, a phenotypic screen only partially covers the mutation combination space, but may still identify a DH line with reduced number of mutations and with the desired phenotype. This stage can be followed up by a complete screen after iteratively narrowing down the number of targets (*g*). When the number of target genes (*g*) is relatively small, a collection of *n* DH lines can cover all possible combinations in a complete phenotypic screen ($n >> 2^g$). (**d**) A second possible goal is to generate and identify in a targeted way a specific, predefined homozygous genotype. For instance, a set of genes for which all homozygous gene edits are desired may be defined based on i) association data; ii) all genes in a QTL region; or iii) a gene family. The minimal population size ($n_{min}$, *i.e.* collection of GEDH lines) required to identify a favorable genotype with a given probability using a DH strategy can be estimated (Lübberstedt & Frei, 2012). Here, we calculated the relationship between the probability to identify a particular genotype for increasing number of targets (*g*), given a feasible population size in four scenarios (146 or 1000 DH lines, or 1000 or 9430 plants obtained after a self-cross).

## References

**Hunter JD. 2007.** Matplotlib: A 2D graphics environment. *Computing in science & engineering* **9:** 90-95.

**Lorenzo CD, Debray K, Herwegh D, Develtere W, Impens L, Schaumont D, Vandeputte W, Aesaert S, Coussens G, De Boe Y*, et al.* 2023.** BREEDIT: a multiplex genome editing strategy to improve complex quantitative traits in maize. *Plant Cell* **35:** 218-238.

**Lübberstedt T, Frei UK. 2012.** Application of doubled haploids for target gene fixation in backcross programmes of maize. *Plant Breeding* **131:** 449-452.

**Stelpflug SC, Sekhon RS, Vaillancourt B, Hirsch CN, Buell CR, de Leon N, Kaeppler SM. 2016.** An expanded maize gene expression atlas based on RNA sequencing and its use to explore root development. *The plant genome* **9:** plantgenome2015.2004.0025.

**Van Huffel K, Stock M, Ruttink T, De Baets B. 2022.** Covering the Combinatorial Design Space of Multiplex CRISPR/Cas Experiments in Plants. *Frontiers in Plant Science***:** 1870.

**Waskom ML. 2021.** Seaborn: statistical data visualization. *Journal of Open Source Software* **6:** 3021.
